# α-Synuclein Pathology in Idiopathic Normal Pressure Hydrocephalus: Evidence for Concomitant Synucleinopathy and Clinical Implications

**DOI:** 10.3390/diagnostics16182889

**Published:** 2026-09-08

**Authors:** Efstratios-Stylianos Pyrgelis, George P. Paraskevas, Vasilios C. Constantinides, Fotini Boufidou, Leonidas Stefanis, Elisabeth Kapaki

**Affiliations:** 1 Neurochemistry and Biological Markers Unit, 1st Department of Neurology, School of Medicine, National and Kapodistrian University of Athens, Eginition Hospital, Vass. Sophias Ave. 74, 11528 Athens, Greece; geoprskvs44@gmail.com (G.P.P.); vassilis.kon@hotmail.com (V.C.C.); fboufidou@med.uoa.gr (F.B.); lstefanis@med.uoa.gr (L.S.); ekapaki@med.uoa.gr (E.K.); 2 2nd Department of Neurology, School of Medicine, National and Kapodistrian University of Athens, “Attikon” University General Hospital, Rimini 1, 12462 Athens, Greece; 3 1st Department of Neurology, School of Medicine, National and Kapodistrian University of Athens, Eginition Hospital, Vass. Sophias Ave. 74, 11528 Athens, Greece

**Keywords:** idiopathic normal pressure hydrocephalus, α-synuclein, biomarkers, cerebrospinal fluid, synucleinopathy, Parkinson’s disease, Alzheimer’s disease, shunt surgery, differential diagnosis, prognosis

## Abstract

Idiopathic normal pressure hydrocephalus (iNPH) is a potentially reversible neurological disorder characterized by gait impairment, cognitive decline, and urinary dysfunction. Despite improvements in diagnostic criteria and neuroimaging techniques, accurate diagnosis remains challenging due to clinical overlap with neurodegenerative diseases, particularly Alzheimer’s disease (AD) and synucleinopathies. Biomarkers capable of improving differential diagnosis, patient selection for shunt surgery, and prognostic assessment are therefore of considerable clinical interest, as has been in AD biomarkers. This review summarizes current evidence regarding the role of α-synuclein as a potential additional marker in iNPH, focusing on its association with concomitant neurodegenerative pathology, clinical manifestations, and treatment outcomes. Available studies indicate that α-synuclein pathology may coexist with iNPH in a substantial proportion of patients, with reported positivity rates ranging from approximately 14% to 33%, depending on the detection method used. Patients with concomitant α-synuclein pathology may exhibit clinical features atypical for pure iNPH, including upper limb rigidity, olfactory dysfunction, hallucinations, autonomic dysfunction, sleep disturbances, and fluctuating cognitive impairment. However, current evidence does not support α-synuclein positivity as an independent predictor of poor response to cerebrospinal fluid (CSF) drainage or shunt surgery. On the contrary, α-synuclein presence may provide additional information regarding mixed neurodegenerative pathology and contribute to personalized clinical management. Future studies should focus on standardized detection techniques, larger multicenter cohorts, longitudinal follow-up, and integration of α-synuclein assessment with established AD biomarkers, neuroimaging findings, and clinical evaluation. Overall, α-synuclein represents a promising complementary marker in iNPH, with potential value in being taken into consideration during clinical evaluation and understanding disease heterogeneity.

## 1. Introduction

Hydrocephalus is characterized by abnormal enlargement of the cerebral ventricles due to disturbances in cerebrospinal fluid (CSF) circulation. It is traditionally classified into obstructive and non-obstructive forms [1]. Normal pressure hydrocephalus (NPH) represents a clinical subtype of non-obstructive hydrocephalus characterized by the classical triad of gait disturbance, cognitive impairment, and urinary dysfunction, despite normal or intermittently elevated CSF pressure [2,3]. When no identifiable secondary cause, such as previous subarachnoid hemorrhage, meningitis, or traumatic brain injury, can be established, the condition is classified as idiopathic normal pressure hydrocephalus (iNPH) mainly occurring in the elderly [3].

The potentially reversible nature of iNPH makes timely diagnosis essential, as appropriate patient selection for CSF diversion procedures, particularly ventriculoperitoneal shunt surgery, can result in significant clinical improvement [2,3]. However, diagnosis remains challenging because the clinical presentation of iNPH frequently overlaps with common neurodegenerative disorders of aging, including Alzheimer’s disease (AD), Parkinson’s disease (PD), and dementia with Lewy bodies (DLB) [4,5]. Furthermore, neuropathological studies suggest that “pure” iNPH may represent only a subset of cases, since concomitant neurodegenerative pathology is frequently observed.

Consequently, the identification of reliable biomarkers has become a major research priority in iNPH. This is true for AD biomarkers which have been shown to improve diagnostic accuracy, facilitate differential diagnosis, assist in the selection of patients likely to benefit from shunt surgery, and provide insight into the underlying mechanisms contributing to disease heterogeneity [6]. Cerebrospinal fluid biomarkers associated with AD pathology, including amyloid-beta 42 (Aβ42), the Aβ42/Aβ40 ratio, phosphorylated tau (p-tau), and total tau (t-tau), have been extensively investigated and may contribute to diagnostic and prognostic assessment in iNPH [6,7,8,9,10].

More recently, α-synuclein has emerged as a potential biomarker of interest. α-Synuclein is a 14 kDa presynaptic neuronal protein expressed predominantly within neurons of the central and peripheral nervous systems, although it is also detectable in peripheral tissues and blood cells. Physiologically, α-synuclein participates in synaptic vesicle trafficking and regulation of neurotransmitter release [11]. Abnormal misfolding and aggregation of α-synuclein result in intracellular inclusions known as Lewy bodies, which represent the pathological hallmark of synucleinopathies, including Parkinson’s disease and dementia with Lewy bodies [11,12]. α-synuclein can be measured in serum, plasma, CSF, saliva, skin and other tissues [13].

Recent investigations have examined CSF α-synuclein concentrations and α-synuclein seed amplification assays (SAAs) in patients with iNPH as potential indicators of neuronal dysfunction, impaired protein clearance, or concomitant synuclein pathology [6]. Given the central role of impaired CSF circulation and glymphatic dysfunction in iNPH pathophysiology, alterations in α-synuclein clearance mechanisms may provide important insights into disease mechanisms and clinical variability.

The assessment of α-synuclein together with established AD biomarkers may therefore improve differentiation between pure iNPH and mixed neurodegenerative conditions. Furthermore, identification of concomitant synucleinopathy may contribute to more individualized therapeutic strategies.

The aim of this review is to summarize and critically evaluate current evidence regarding α-synuclein as a potential additional marker in iNPH, focusing on its role in differential diagnosis, association with clinical phenotypes, and prognostic implications following CSF drainage and shunt surgery. Thus, the novelty of this review is not based only on extending this concept from AD to α-synuclein pathology. We rather aim to provide a focused conceptual synthesis of the current evidence regarding the potential clinical and pathophysiological significance of α-synuclein pathology in iNPH.

## 2. Materials and Methods

**Scheme 1:**
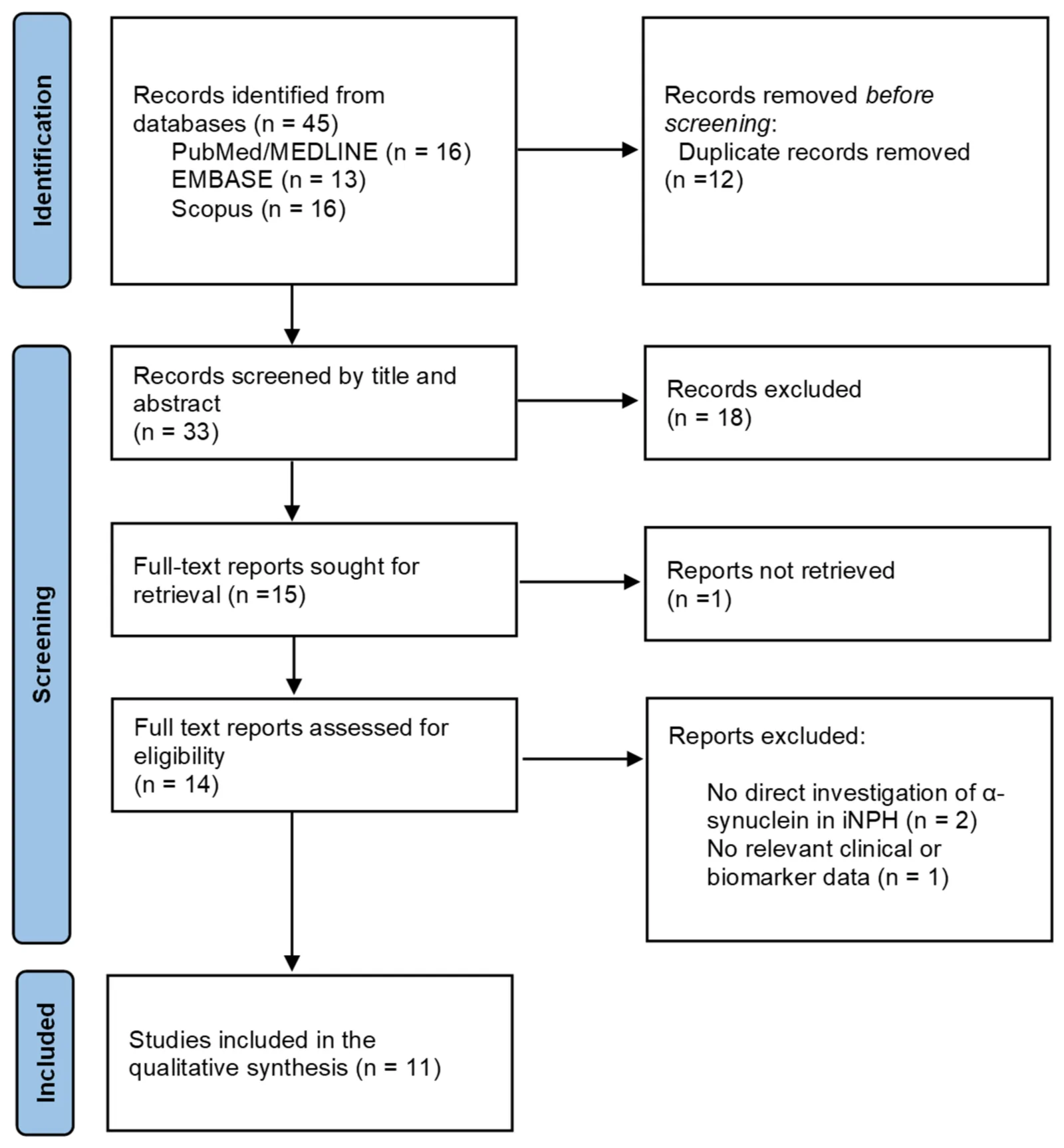
Studies’ selection flow diagram.

A comprehensive literature search was conducted in PubMed/MEDLINE, EMBASE, and Scopus from database inception to 10 June 2026, with no restrictions regarding publication date or language. Keywords used to query the database included: “idiopathic normal pressure hydrocephalus (iNPH)”, “α-synuclein”, “biomarkers”, and prion seed amplification assays, including “RT-QuIC,” “real-time quaking-induced conversion,” “seed amplification assay,” “prion amplification,” and related terms. The Boolean operators “AND” and “OR” were used to combine search concepts, and the search strategy was adapted to the specific indexing requirements of each database. Studies were considered eligible if they investigated α-synuclein or related biomarkers in patients with iNPH and provided relevant clinical, diagnostic, or biomarker data. Editorials, case reports, case series, reviews, and case–control and cohort studies were also evaluated, and relevant information was selected. Duplicate publications were excluded from further assessment. Reference lists of all articles that met the criteria and references of relevant review articles were examined to identify studies that may have been missed by the database search. The study selection process, including identification, screening, eligibility assessment, and final inclusion, was performed by two reviewers and is documented using a PRISMA flow diagram (Scheme 1). When disagreements occurred during the selection process, reviewers discussed the study against predefined inclusion criteria to reach consensus. Relevant data were extracted from the included studies and subsequently summarized and synthesized qualitatively. Generative artificial intelligence (GenAI) has been solely used to assist in the figures design.

## 3. Results

Main characteristics of recent publications based on original studies focusing on α-synuclein and iNPH are depicted in Table 1.

### 3.1. Clinical Manifestations Suggestive of Concomitant α-Synuclein Pathology in iNPH

The relationship between iNPH and α-synucleinopathy represents an expanding area of investigation. Although gait impairment in iNPH has traditionally been attributed to mechanical distortion of periventricular white matter structures and corticospinal pathways caused by ventricular enlargement, this mechanism alone does not fully explain the presence of parkinsonian features observed in a proportion of patients.

Functional neuroimaging studies have demonstrated alterations in cerebral perfusion and connectivity within cortico-basal ganglia-thalamo-cortical networks and frontal motor circuits, suggesting that dysfunction of these pathways contributes to gait impairment and the development of a parkinsonian phenotype in iNPH [22].

The typical gait disorder of iNPH is characterized by slow, broad-based, unstable steps with reduced step height and a “magnetic” quality [23,24]. Although there are some clinical differences between iNPH-related gait impairment and Parkinsonian gait disturbances, sometimes it could be rather challenging to distinguish between the two conditions. Patients with isolated iNPH generally do not exhibit classic Parkinson’s disease features such as resting tremor, reduced arm swing, marked rigidity, or camptocormia [25].

However, the presence of additional clinical manifestations may suggest concomitant α-synuclein pathology. Recent studies have reported that iNPH patients with α-synuclein positivity more frequently demonstrate upper limb rigidity, impaired olfaction, hallucinations, autonomic dysfunction, sleep disturbances, and fluctuating cognitive impairment compared with α-synuclein-negative patients [18,20].

Bulbar symptoms, including dysphagia, dysarthria, hypomimia, and sialorrhea, may occur in iNPH but appear to be more strongly associated with Parkinsonian syndromes when present together with other suggestive manifestations [26,27]. Similarly, neuropsychiatric symptoms such as apathy, depression, and anxiety may occur in both iNPH and synucleinopathies and therefore have limited specificity when considered individually [16]. The symptoms that are suggestive of coexistence of α-synucleinopathy in iNPH patients are summarized in Table 2.

Overall, no single clinical feature is sufficiently specific to diagnose concomitant α-synucleinopathy in iNPH. Instead, the presence of a characteristic symptom pattern should prompt additional biomarker investigation.

### 3.2. Coexistence of Idiopathic Normal Pressure Hydrocephalus with Alzheimer’s Disease Pathology and α-Synucleinopathy

**Figure 1:**
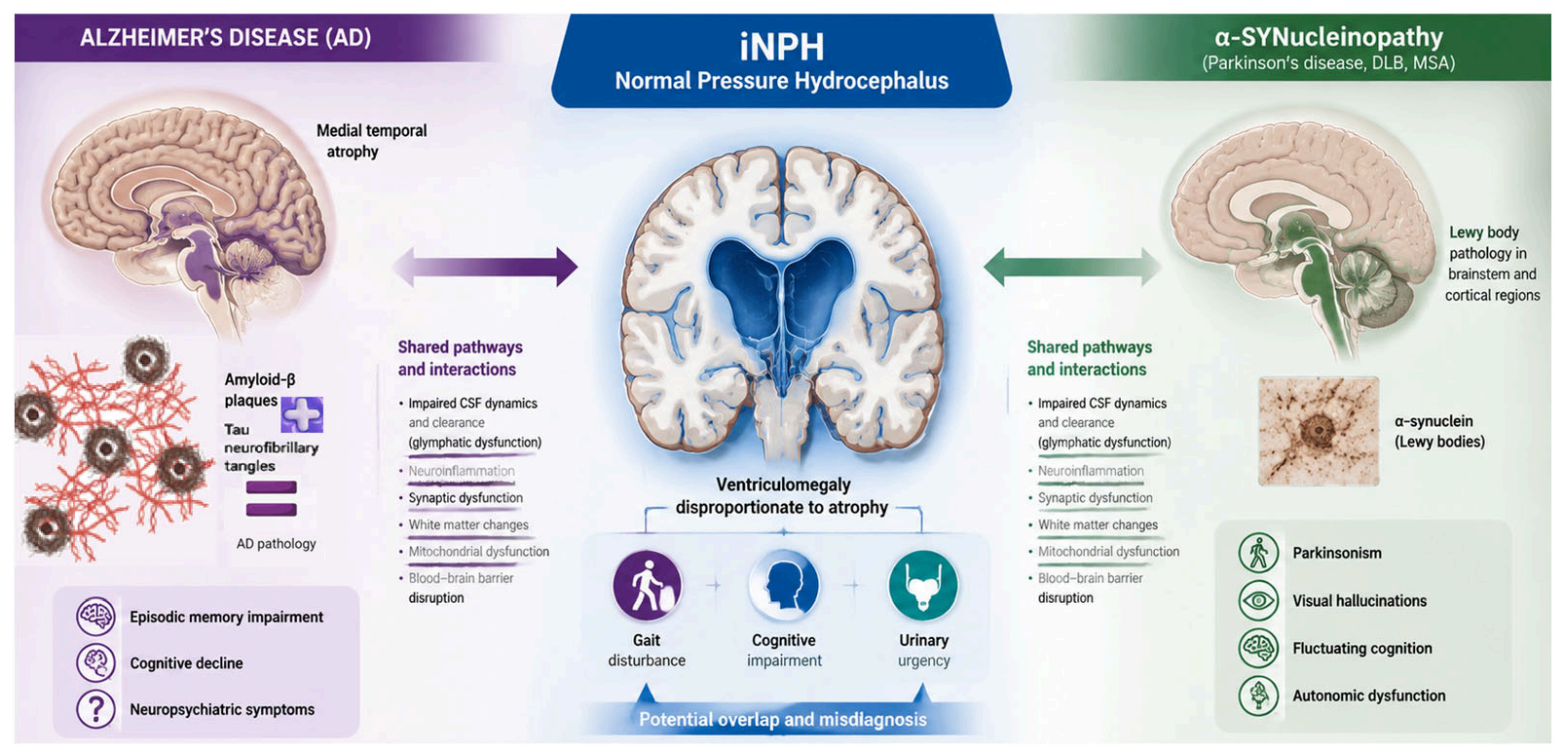
The relation of AD pathology and a-synucleinopathy with iNPH.

One of the major challenges in the diagnosis and clinical management of idiopathic normal pressure hydrocephalus (iNPH) is the frequent coexistence of neurodegenerative pathology. The clinical manifestations of iNPH overlap substantially with those of Alzheimer’s disease (AD) and synucleinopathies, particularly Parkinson’s disease (PD) and dementia with Lewy bodies (DLB), making the identification of underlying pathological processes difficult based solely on clinical assessment (Figure 1).

Neuropathological and biomarker studies indicate that concomitant AD pathology is relatively common among patients with iNPH, with reported prevalence rates ranging from approximately 19% to 68% depending on the population studied and the methodology used for detection [28]. Similarly, recent studies investigating α-synuclein pathology suggest that a significant subgroup of iNPH patients may harbor concomitant synucleinopathy. Using cerebrospinal fluid (CSF) α-synuclein seed amplification assays (SAAs), available studies have reported α-synuclein positivity in approximately 14–33% of patients with iNPH; however, these estimates derive from heterogeneous study populations and methodologies and should not be interpreted as a definitive prevalence in the iNPH population [16,17,19,20].

The variability in reported prevalence is likely influenced by differences in detection methods. CSF-based α-synuclein SAAs detect pathological seeding activity in soluble biological samples, whereas peripheral tissue approaches, such as skin biopsy with immunofluorescence techniques, identify pathological α-synuclein deposits directly within peripheral nerves. Some studies suggest that skin biopsy may demonstrate higher sensitivity compared with CSF-based approaches, although direct comparisons in pathologically verified cohorts remain limited [29].

Recent literature data has further highlighted the frequency of mixed pathology in iNPH. Wluka et al. reported that a substantial proportion of patients undergoing biomarker assessment demonstrated evidence of either AD pathology, α-synuclein pathology, or both, supporting the concept that iNPH frequently represents a heterogeneous clinical syndrome rather than a single pathological entity [20].

The presence of concomitant neurodegenerative pathology may contribute to the variability observed in clinical presentation, cognitive profile, and treatment response among patients with iNPH. Therefore, biomarkers such as α-synuclein may provide clinically relevant information by identifying patients with mixed pathological processes who may require a more individualized diagnostic and therapeutic approach.

### 3.3. α-Synuclein as a Potential Prognostic Marker in Idiopathic Normal Pressure Hydrocephalus

In contrast to Alzheimer’s disease (AD) cerebrospinal fluid (CSF) biomarkers, there is less evidence available regarding the prognostic significance of α-synuclein pathology in patients with idiopathic normal pressure hydrocephalus (iNPH). A clinically important question is whether the presence of concomitant α-synuclein pathology influences the response to CSF drainage procedures or shunt surgery.

Recent studies have investigated whether α-synuclein-positive and α-synuclein-negative patients demonstrate different clinical outcomes following CSF drainage. Fasano et al. reported that α-synuclein-negative patients showed greater improvement in postural stability following CSF drainage compared with α-synuclein-positive patients. However, improvements in gait velocity and step length were comparable between the two groups, suggesting that α-synuclein pathology may have limited influence on the overall motor response to CSF removal [16].

Similarly, Yu et al. investigated long-term outcomes following ventriculoperitoneal shunt surgery and found that patients with iNPH experienced sustained clinical improvement regardless of α-synuclein status. These findings suggest that the presence of α-synuclein pathology should not automatically exclude patients from surgical treatment when other diagnostic criteria support the diagnosis of iNPH [19].

Giannini et al. (2022) found that α-synuclein positivity does not significantly correlate with clinical outcome after shunt surgery [17]. More recently, Wluka et al. evaluated the clinical utility of incorporating α-synuclein seed amplification assays into the diagnostic assessment of iNPH. Their findings demonstrated no significant differences in postoperative outcomes between α-synuclein-positive and α-synuclein-negative patients. However, the authors suggested that patients with persistent parkinsonian symptoms despite adequate CSF drainage may benefit from additional evaluation for concomitant Parkinson’s disease and consideration of a levodopa trial when clinically appropriate [20]. The main findings regarding the potential prognostic role of α-synuclein pathology in iNPH are summarized in Table 3.

Overall, current evidence suggests that α-synuclein positivity does not represent a contraindication to shunt surgery. Instead, α-synuclein presence may provide additional information regarding underlying disease complexity and the possibility of mixed neurodegenerative pathology.

## 4. Discussion

This review highlights the emerging role of α-synuclein as a potential complementary marker in idiopathic normal pressure hydrocephalus (iNPH), particularly for identifying concomitant synucleinopathy and improving understanding of disease heterogeneity. Although current evidence does not support α-synuclein as an independent diagnostic or prognostic marker, its assessment may provide clinically meaningful information regarding underlying neurodegenerative processes in a subgroup of patients.

A major finding from recent studies is the high prevalence of mixed neurodegenerative pathology among individuals diagnosed with iNPH. AD-related pathology has been reported in approximately one-fifth to two-thirds of patients, while α-synuclein pathology has been detected in approximately 14–33% of cases depending on the detection method used [16,17,19,20]. These observations reinforce the concept that iNPH frequently coexists with other age-related neurodegenerative disorders and that clinical heterogeneity may partly reflect differences in underlying pathology.

An important distinction should be made between conventional assays measuring total α-synuclein concentrations and the more recent α-synuclein seed amplification assays. Earlier studies predominantly relied on immunoassays such as ELISA to quantify total α-synuclein in biological fluids. However, total α-synuclein measurements have shown substantial variability and limited diagnostic specificity, possibly because they quantify the total protein measurement without specifically distinguishing physiological, soluble α-synuclein from pathological misfolded or aggregated a-synuclein. On the other hand, modern α-synuclein amplification assays, including real-time quaking-induced conversion (RT-QuIC) and protein misfolding cyclic amplification (PMCA), take into account the seeding properties of pathological α-synuclein to amplify amounts of misfolded aggregates, thereby providing a fundamentally different measurement which has pathophysiological meaning [30]. This methodological distinction is particularly important when interpreting the high diagnostic specificity reported in recent studies. Rather than reflecting simply an increased ability to quantify α-synuclein, the high specificity of recent techniques is largely related to their ability to detect misfolded, aggregation-prone α-synuclein seeds, which are closely linked to the underlying synucleinopathy.

The introduction of α-synuclein seed amplification assays has substantially improved the ability to detect pathological α-synuclein aggregation in biological samples. However, methodological variability remains a major limitation. Differences between CSF-based assays, skin biopsy techniques, and other detection platforms may contribute to inconsistent prevalence estimates across studies. Standardization of analytical protocols and direct comparison of different methodologies within the same patient cohorts will be essential before α-synuclein testing can be routinely implemented in clinical practice.

From a clinical perspective, α-synuclein-positivity in iNPH appears to be connected with increased frequency of features associated with synucleinopathies. These include upper limb rigidity, impaired olfaction, hallucinations, autonomic dysfunction, sleep abnormalities, and fluctuating cognitive impairment [18,20]. However, none of these findings alone is sufficiently specific to establish the diagnosis of concomitant synucleinopathy. Instead, the combination of clinical features and biomarker findings may provide a more accurate representation of the underlying disease process.

The coexistence of α-synuclein pathology also provides important insights into the pathophysiology of iNPH. Traditionally, gait impairment has been attributed primarily to ventricular enlargement and mechanical disruption of periventricular structures. However, this explanation does not fully account for the presence of extrapyramidal symptoms in many patients. Alterations in cortico-basal ganglia-thalamo-cortical networks and frontal motor pathways may contribute to gait dysfunction and parkinsonian manifestations [22]. In this context, concomitant α-synuclein aggregation may represent an additional pathological mechanism contributing to motor and cognitive symptoms.

Furthermore, impaired CSF circulation and glymphatic dysfunction, which are increasingly recognized components of iNPH pathophysiology, may influence the clearance of misfolded proteins. In iNPH, impaired CSF circulation and clearance as long as progressive ventriculomegaly may disrupt the normal exchange between CSF and interstitial fluid. These alterations have been associated with delayed clearance of CSF tracers and impaired glymphatic function, suggesting that iNPH is characterized not simply by ventricular enlargement but by wider disturbances in brain-fluid homeostasis. Aquaporin-4 (AQP4), which is highly expressed in astrocytic surrounding cerebral blood vessels, is considered an important component of the glymphatic pathway. Notably, neuropathological studies in iNPH have demonstrated reduced perivascular AQP4 expression, potentially impairing the movement and clearance of interstitial solutes. More recent neuroimaging evidence further supports this association, showing that glymphatic dysfunction assessed using the diffusion tensor imaging analysis along the perivascular spaces (DTI-ALPS) index is present in iNPH and is inversely associated with ventricular volume. Together, these findings suggest that ventriculomegaly and altered CSF dynamics may contribute to a self-reinforcing cycle of impaired perivascular fluid transport and reduced clearance of potentially neurotoxic proteins [31,32]. Reduced clearance of α-synuclein may theoretically promote its accumulation within the central nervous system, creating a possible mechanistic link between CSF dysregulation and neurodegenerative proteinopathies. However, this hypothesis requires further experimental and longitudinal validation.

The prognostic implications of α-synuclein pathology remain less clearly defined. Unlike AD biomarkers, which have been associated with reduced postoperative benefit in several studies, current evidence suggests that α-synuclein positivity does not substantially impair response to CSF drainage or shunt surgery [16,19,20]. Therefore, identification of α-synuclein pathology should not be interpreted as evidence against surgical treatment. Instead, it may help clinicians anticipate additional neurological manifestations and optimize long-term management strategies.

Several limitations characterize the current evidence base. Most available studies include relatively small cohorts, originate from single centers, are retrospectively designed and use heterogeneous diagnostic approaches. Differences in inclusion criteria, α-synuclein detection methods and outcome measurements limit direct comparison between studies. Furthermore, longitudinal studies evaluating whether α-synuclein positivity could predict future cognitive decline, development of parkinsonism, or long-term treatment response remain rare.

The clinical utility of α-synuclein testing in iNPH remains to be fully established. Although α-synuclein positivity may identify patients with concomitant synucleinopathy and thereby provide additional diagnostic information, current evidence does not demonstrate that it meaningfully predicts response to CSF drainage or shunt surgery, which remains the key therapeutic decision in iNPH. Thus, α-synuclein should currently be viewed primarily as a biomarker of underlying synucleinopathy rather than as a definitive biomarker for iNPH diagnosis, prognosis, or treatment selection.

Future research should focus on prospective multicenter studies incorporating standardized α-synuclein detection protocols alongside established AD biomarkers, neuroimaging, and detailed clinical phenotyping. A multimodal biomarker approach is likely to provide greater diagnostic accuracy than reliance on a single pathological marker. Longitudinal investigations examining interactions between CSF dynamics, glymphatic function, protein clearance, and neurodegenerative progression will also be essential.

## 5. Conclusions

Current evidence suggests that α-synuclein represents a promising complementary marker in idiopathic normal pressure hydrocephalus rather than a disease-specific diagnostic biomarker. Its primary clinical value appears to be the identification of patients with concomitant synucleinopathy, thereby improving differential diagnosis and potentially assisting in better stratification of patients regarding other than shunting therapeutic approaches. At present, α-synuclein positivity should not be considered a contraindication to CSF diversion procedures, as available evidence indicates that many α-synuclein-positive patients continue to achieve meaningful improvement following shunt surgery. Future integration of α-synuclein biomarkers with Alzheimer’s disease biomarkers, neuroimaging findings, and comprehensive clinical assessment may enable a more precise biological classification of iNPH and facilitate personalized therapeutic strategies.

## Figures and Tables

**Table 1:** Features of studies focusing on α-synuclein and iNPH.

Study’s Authors- Year of Publication	Total Cohort’s Size	Inclusion Criteria	Cohort’s Characteristics	Type of Study/ Modality Used for α-Synuclein Measurement
Mollenhauer et al., 2012 [14]	47	Patients with normal white blood cell count, absence of intrathecal immunoglobulin production, normal CSF tau protein, and no LP-induced contamination by blood	2 sub-cohorts from different centers: 42 with various neurological disorders (**4 with iNPH**) **5 with iNPH**	Prospective study α-synuclein and serum α-synuclein (diluted at 1:15) concentrations were measured by ELISA
Sakurai et al., 2022 [15]	79	Patients who met the criteria for both probable iNPH and definite iNPH. Patients whose CSF showed Aβ42 < 500 pg/mL, total tau > 600 pg/mL, or phosphorylated tau > 60 pg/mL were excluded. Clinically established or clinically probable PD diagnosis was based on the MDS-PD criteria. Patients with DLB met the criteria for probable DLB from the DLB Consortium	**34 iNPH without** **accompanying** **disorders** **23 iNPH with comorbid PD/PDD** **8 iNPH with comorbid DLB**	Retrospective study Implementation of α-synuclein RT-QuIC in CSF
Fasano et al., 2022 [16]	50	iNPH patients were categorized as “possible” iNPH in case of radiological evidence of hydrocephalus and at least one clinical sign, “probable” with the addition of disproportionately enlarged subarachnoid space and gait impairment improved after lumbar drainage, and definite with the addition of improvement after shunt surgery	**50 patients with iNPH** divided into LP responders and non-responders	Retrospective study Implementation of α-synuclein RT-QuIC in CSF
Giannini et al., 2022 [17]	293	iNPH diagnosis was based on consensus guideline criteria and all patients fulfilled the criteria for probable iNPH	**293 patients with iNPH** from two independent cohorts	Retrospective study Implementation of α-synuclein RT-QuIC in CSF
Weber et al., 2025 [18]	144	Diagnosis of iNPH, according to established Criteria. Patients with the diagnosis of PD or DLB according to diagnostic criteria were excluded	**144 iNPH patients** from a single center, Patients with PD or DLB were excluded	Retrospective study Implementation of α-synuclein -SAA in CSF
Yu et al., 2025 [19]	33	Patients were diagnosed with “probable” iNPH, using the following criteria: age > 40 years, symptoms insidious development over 6 months, normal CSF opening pressure, enlarged ventricles and no macroscopic obstruction of CSF flow. The diagnosis was confirmed by Evans’ index over 0.30 and at least one of: acute callosal angle; aqueductal or fourth ventricular flow void and disproportionately enlarged subarachnoid spaces.	**33 iNPH patients** without evidence of nigrostriatal degeneration	Retrospective study Implementation of α-synuclein RT-QuIC in CSF
Wluka et al., 2026 [20]	89	The differential diagnosis includes AD, PD and DLB. iNPH typically presents with gait disturbance before the onset of cognitive decline. When cognitive impairment is the predominant concern and gait is minimally disturbed, additional testing was done to exclude AD or DLB. Marked memory deficits with relatively preserved executive function strengthens the suspicion for AD.	**89 iNPH patients**	Prospective study Implementation of α-synuclein skin biopsy test
Dinter et al., 2026 [21]	356	Criteria included inter national consensus criteria for DLB, MDS criteria for PD, MSA and PSP, Gold Coast diagnostic criteria for ALS	90 patients with Parkinsonian Syndromes 139 with predominant cognitive disorders 25 with other movement disorders 35 with inflammatory or (para)neoplastic syndromes, 67 with further diseases **14 with iNPH**	Cross-sectional study Implementation of binary α-synuclein -SAA in CSF

**Table 2:** Clinical features associated with possible concomitant α-synuclein pathology in patients with iNPH.

Clinical Feature	Typical Presentation in iNPH	Findings Suggestive of Concomitant α-Synuclein Pathology	Reference(s)
Gait disturbance	Slow, unstable, broad-based “magnetic” gait	Progressive parkinsonian gait features or atypical motor progression	[23,24,25]
Upper limb rigidity	Usually absent or mild	More frequent in α-synuclein-positive patients	[18]
Bulbar symptoms	Possible but uncommon	Increased frequency may suggest synucleinopathy	[26,27]
Olfactory dysfunction	Not a typical feature	Reduced olfactory function associated with α-synuclein pathology	[18]
Hallucinations	Uncommon in isolated iNPH	Increased occurrence suggests Lewy body spectrum pathology	[18]
Autonomic dysfunction	Not a core diagnostic feature	Orthostatic hypotension may indicate synucleinopathy	[20]
Sleep Disturbances	May occur	Suggestive when combined with other synucleinopathy features	[20]
Cognitive Impairment	Core feature of iNPH	Fluctuating or unstable cognition may indicate mixed pathology	[20]
Neuropsychiatric symptoms	Apathy, depression, anxiety may occur	Supportive but nonspecific findings	[16]

**Table 3:** Studies evaluating the prognostic role of α-synuclein pathology in iNPH.

Study	Methodology	Positivity of α-Synuclein in iNPH Patients	Main Findings
Fasano et al., 2022 [16]	Assessment of α-synuclein status and response to CSF drainage	14%	α-Synuclein-negative patients demonstrated greater improvement in standing balance; gait velocity and step length improved similarly in both groups.
Giannini et al., 2022 [17]	Integration of α-synuclein and AD biomarkers in iNPH evaluation	20.5%	No significant difference in shunt outcomes between α-synuclein-positive and α-synuclein-negative patients; levodopa trial may be useful in positive ones
Yu et al., 2025 [19]	Evaluation of α-synuclein status and outcomes after ventriculoperitoneal shunt surgery	33.3%	Durable clinical improvement occurred regardless of α-synuclein positivity status.
Wluka et al., 2026 [20]	Integration of α-synuclein and AD biomarkers in iNPH evaluation	31%	No significant difference in shunt outcomes between α-synuclein-positive and α-synuclein-negative patients; levodopa trial may be considered in selected patients with persistent parkinsonism.

## Data Availability

No new data were created or analyzed in this study. Data sharing is not applicable to this article.

## Abbreviations

The following abbreviations are used in this manuscript:

iNPH	Idiopathic normal pressure hydrocephalus
CSF	Cerebrospinal fluid
AD	Alzheimer’s Disease
PD	Parkinson’s Disease
DLB	Dementia with Lewy bodies
SAA	seed amplification assay
αSyn	α-Synuclein
RT-QuIC	Real-Time Quaking-Induced Conversion
ELISA	Enzyme-linked Immunosorbent Assay
